# Case report: Laryngospasm following ethanol ablation of a parathyroid nodule in a dog with primary hyperparathyroidism

**DOI:** 10.3389/fvets.2023.1201663

**Published:** 2023-06-15

**Authors:** Kaitlyn Rank, Alex M. Lynch, Randolph Green, Leslie Reed-Jones, Karyn Harrell, Yu Ueda

**Affiliations:** Department of Clinical Sciences, College of Veterinary Medicine, North Carolina State University, Raleigh, NC, United States

**Keywords:** iatrogenic, hyperparathyroidism, hypocalcemia, ethanol ablation, laryngospasm

## Abstract

A 12-year-old female spayed dachshund was presented for emergency assessment of respiratory distress, characterized by inspiratory dyspnea with stridor. Percutaneous ultrasound-guided ethanol ablation of a functional parathyroid tumor was performed 72-h earlier for management of primary hyperparathyroidism. The dog was hypocalcemic (ionized calcium 0.7 mmol/L, reference interval: 0.9–1.3 mmol/L) at the time of presentation and had evidence of laryngospasm on a sedated oral exam. The dog was managed conservatively with supplemental oxygen, anxiolysis, and parenteral calcium administration. These interventions were associated with rapid and sustained improvement in clinical signs. The dog did not demonstrate any recurrence of signs afterwards. To the authors' knowledge, this is the first description of laryngospasm following ethanol ablation of a parathyroid nodule in a dog that developed hypocalcemia.

## Introduction

Primary hyperparathyroidism is a rare condition in dogs, which develops secondary to parathyroid adenomas, parathyroid carcinomas, or parathyroid hyperplasia (1). Common clinical signs include those relating to hypercalcemia, including polyuria, polydipsia, lethargy, weakness, hyporexia, vomiting, and diarrhea (1). Between 24%−29% of affected dogs develop clinical signs related to urolithiasis, including hematuria, dysuria, stranguria, and pollakiuria (1). Most confirmed cases are treated with surgical removal of the parathyroid gland (2). Hypercalcemia usually resolves within 6 days of surgery and has a low recurrence rate, although up to 40% of dogs are reported to develop hypocalcemia in the postoperative period (1). Alternative approaches to surgery include ultrasound-guided ethanol or heat ablation of a parathyroid nodules (1–3). Hypercalcemia resolved in 85% of dogs after ethanol ablation, with resolution occurring within 72 h in 96% of cases (1). Hypocalcemia was reported to occur in 22% of this cohort of patients, with 1 out of 6 patients (16%) developing symptomatic hypocalcemia requiring treatment. The most common signs of hypocalcemia are neuromuscular in dogs, including tremors and tetany. Overall, the complication rate for ethanol ablation of parathyroid tumors is reported to be 11% (3). Apart from hypocalcemia, additional adverse effects reported include both temporary and permanent bark changes and coughing associated with laryngeal dysfunction. This likely occurs secondary to chemical disruption or trauma of the recurrent laryngeal nerve. This case report describes a dog with hyperparathyroidism that developed respiratory distress 72 h after ethanol ablation of a parathyroid nodule due to laryngospasm associated with hypocalcemia (4–7).

## Case description

A 12-year-old, 8.1 kg, female spayed dachshund was presented to the emergency service at North Carolina State University College of Veterinary Medicine for assessment of restlessness and respiratory distress. Approximately 72 h before presentation, a percutaneous ultrasound-guided ablation of parathyroid nodule was performed for management of primary hyperparathyroidism. The dog was identified to be hypercalcemic 3 months earlier during diagnostic evaluation of new polyuria/polydipsia signs. Primary hyperparathyroidism was subsequently diagnosed based on recognition of an increased ionized calcium (iCa) [1.78 mmol/L (reference interval (RI): 0.9–1.3)] with increased serum parathyroid hormone concentration [45.2 pmol/L (RI: 1.1–10.6 pmol/L)] and undetectable parathyroid hormone related protein. Ultrasound was performed to better characterize the thyroid and parathyroid glands, and identified a well-defined, rounded, hypoechoic nodule within the cranial pole of the left thyroid gland, consistent with a parathyroid nodule. The dog was previously diagnosed with ACVIM Stage B1 myxomatous mitral valve disease based upon echocardiographic findings (8), for which cardiac medications were not recommended, and hypercortisolism for which trilostane was prescribed (3.8 mg/kg PO q12h; Dechra, Leawood, KS).

The owners elected to pursue percutaneous ultrasound-guided ethanol ablation of the parathyroid nodule instead of surgery to avoid lengthy anesthesia. The dog had a normal physical examination prior to anesthesia, without any signs of respiratory compromise, and normal laryngeal function at the time of endotracheal intubation. Complete blood count and serum biochemistry panels were performed prior to anesthesia. These identified a normal white blood cell count 6.2 × 10^3^/μl (RI: 4.36–11.9 × 10^3^/μl), red blood cell count 8.44 × 10^6^/μl (RI: 5.68–8.80 × 10^6^/μl), and platelet count 296,000 (RI: 190,000–268,000), with mildly elevated plasma protein 8.0 g/dl (RI: 6.1–7.5 g/dl); and moderate total hypercalcemia 15.1 mg/dl (RI: 9.5–11.1 mg/dl), mild hypophosphatemia 2.1 mg/dl (RI: 2.6–5.3 mg/dl), mild hyperalbuminemia 5.1 g/dl (RI: 2.3–4.3 g/dl), moderately elevated alkaline phosphatase 2981 iU/L (RI: 9–88 iU/L) and alanine transaminase 182 iU/L (RI: 17–78 iU/L), and mildly elevated creatine kinase 269 iU/L (RI: 51–255 iU/L). The serum magnesium 2.2 mg/dl (RI: 1.9–2.5 mg/dl) and potassium 4.9 mmol/L (RI: 3.6–5.3 mmol/L) were normal. A normal serum thyroid hormone (T4) was observed 2.5 μg/dl (RI: 1.0–4.0 μg/dl) as part of her initial workup, and her previously diagnosed hypercortisolism was well-controlled as evidenced by an appropriate ACTH stimulation test [pre-ACTH cortisol: 2.2 (RI: 1–5), post-ACTH cortisol: 2.8 (RI: 1.5–6)].

Ultrasound guided ethanol ablation involved injection of 0.56 ml of 95% ethanol (Henry Schein Animal Health, Fort Worth, TX) using a 27-gauge 1.5-inch needle into the parathyroid nodule utilizing a lateromedial approach as previously described (1, 2). A scant volume of fluid outside of the thyroid gland was noted with ultrasound immediately following the procedure, but otherwise no immediate complications were seen. The dog was discharged from the hospital later that day. The dog was described as initially normal by the owner, although developed some behavioral signs of agitation and increased respiratory rate at home 24-h later. The dog was re-examined 48-h after the ablation for a scheduled iCa recheck, at which point a low normal concentration was documented [0.90 mmol/L (RI: 0.9–1.3 mmol/L)]. The dog was next seen 12 h later for assessment of restlessness and tachypnea and increased respiratory effort at home. At the time of presentation, the dog was panting and was diagnosed with a left sided anal sac abscess. The dog was administered 0.2 mg/kg IV of methadone (Akorn Pharmaceuticals, Lake Forest, IL) and 2 mg/kg IV alfaxalone (Jurox Animal Health, Kansas City, MO) to facilitate lancing and lavage of the abscess. Mild hypocalcemia was noted at this time [0.82 mmol/L (RI: 0.9–1.3 mmol/L)] but no specific treatment was started since the dog's clinical signs were attributed to the anal sac abscess not hypocalcemia. The dog was discharged with amoxicillin-clavulanate (15 mg/kg PO q12h; Dechra, Leawood, KS) and gabapentin (12.5 mg/kg PO q8h; Alkem Laboratories, Fenton, MO).

**Figure 1:**
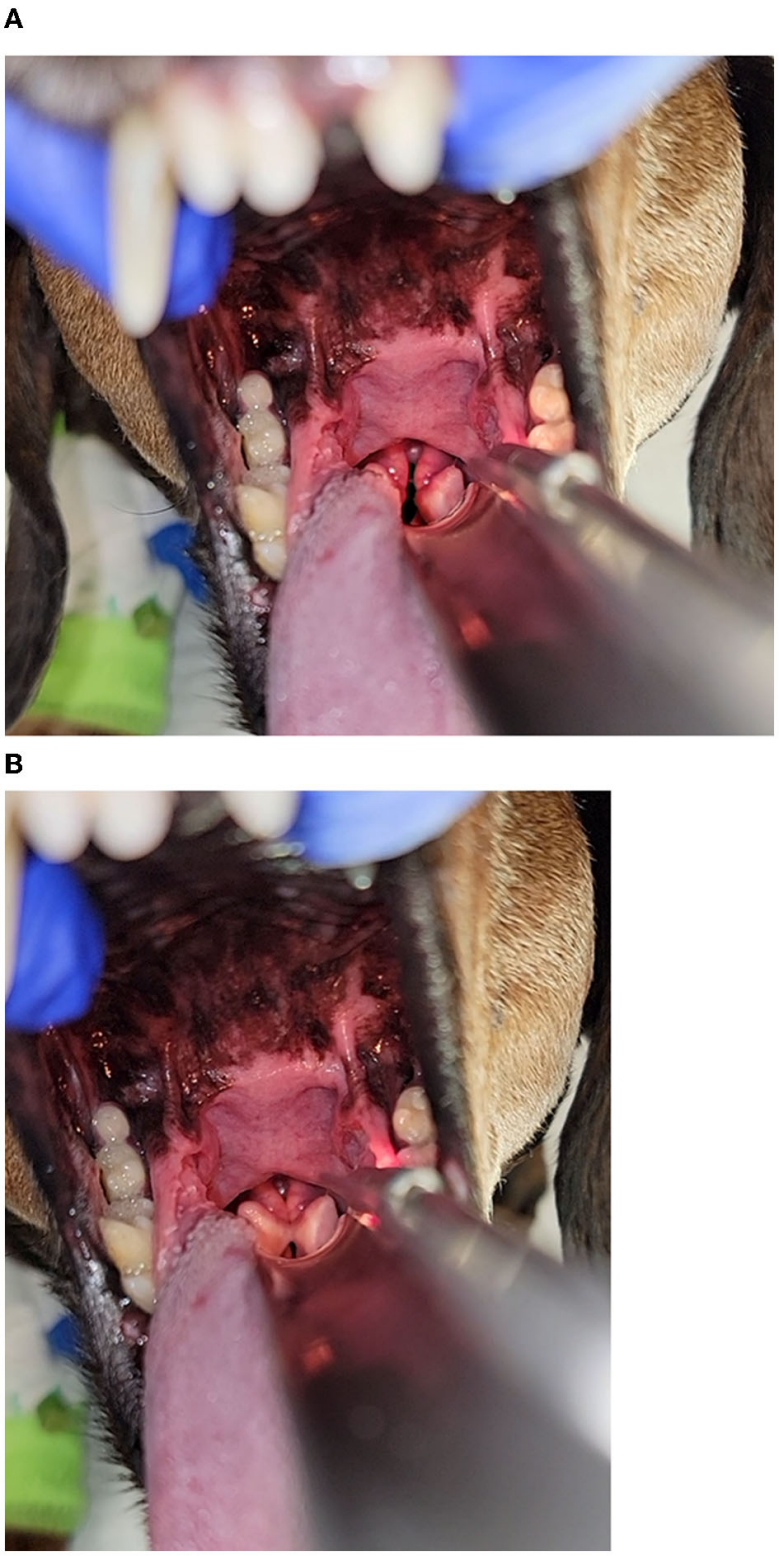
**(A, B)** Images of the larynx obtained under a light plane of anesthesia following the administration of butorphanol and alfaxalone in the dog 72 h after percutaneous ultrasound guided ethanol ablation of the parathyroid nodule. Inappropriate laryngeal movement was noted, including minimal movement of the arytenoid cartilages on inspiration **(A)** and complete closure of the cartilages on expiration **(B)**. No significant perilaryngeal edema or secretions obstructing the airway were visualized. Both left and right sides of the larynx were able to move, albeit minimally, and was most consistent with laryngospasm.

The dog continued to exhibit evidence of respiratory distress and discomfort at home including increased panting, difficulty settling, and restlessness. The owner described the breathing character as “difficulty getting air in, but not out,” which prompted an additional presentation to the emergency service ~20 h later. At the time of evaluation, the dog had evidence of mild tachypnea, respiratory rate 30 breaths/min, characterized by a prolonged inspiratory phase and inspiratory stridor, suggestive an obstructive breathing pattern affecting the larynx; that was accompanied by restlessness and anxious behavior. No evidence of stertor, sneezing, coughing, or retching were noted. Baseline patient data identified a rectal temperature of 39.2°C (102.5°F) and heart rate of 130 beats/min with pink mucous membrane and a capillary refill time of >2 s. Physical examination was normal, except for the previously diagnosed anal sac abscess, and behavioral signs consistent with anxiety. The dog was administered butorphanol (0.3 mg/kg IV; Zoetis Animal Health, Parsippany, NJ). Pulse oximetry indicated an oxygen saturation of 100% and point-of-care thoracic ultrasound revealed a left atrium: aorta ratio of 1.5:1 with rare B-lines on the right hemithorax. Thoracic radiographs were obtained, demonstrating equivocal cardiomegaly, moderate hepatomegaly, and no airway or pulmonary changes to explain her respiratory embarrassment. Alfaxalone was titrated (total dose of 0.5 mg/kg IV) to facilitate a sedated upper airway examination at a plane of anesthesia that preserved swallowing reflexes. Inappropriate laryngeal movement was noted, including minimal movement of the arytenoid cartilages on inspiration with incomplete abduction of the cartilages, and complete closure of the cartilages on expiration. No significant perilaryngeal edema or secretions obstructing the airway were visualized. Both left and right sides of the larynx were able to move, though minimal, making the findings of the airway exam most consistent with laryngospasm (Figures 1A, B, Supplementary Video 1).

A venous blood gas (Gem Premier 5000, Werfen, Bedford, MA) was performed concurrent to this, which demonstrated normal acid base status based on a pH of 7.42 (RI: 7.30–7.48), *P*CO_2_ 32 mmHg (RI: 32–52), and HCO_3_ 20.8 mmol/L (RI: 20.7–29.2), and progressively lower iCa [0.7 mmol/L (RI: 0.9–1.3 mmol/L)]. The dog recovered well from sedation and was initially managed with oral trazodone for anxiolysis (6.3 mg/kg PO q8h; Pliva Inc, East Hanover, NJ). Signs of anxiety and progressive respiratory distress persisted, which prompted initiating supplemental oxygen delivered in a heat-and-humidity controlled cage and a butorphanol constant rate infusion (CRI; 0.2 mg/kg/h IV). Calcium supplementation was also provided (50 mg/kg IV of calcium gluconate 10%; Fresenius Kabi, Lake Zurich, IL) to mitigate any contribution of hypocalcemia to the dog's signs. Shortly afterwards, the dog demonstrated improvement in her respiratory rate and effort, and within 2 h supplemental oxygen and the butorphanol infusion were discontinued. The dog was also managed with maintenance fluid therapy (45 ml/kg/day of 0.45% NaCl; Dechra, Leawood, KS); maropitant (1 mg/kg IV q24h; Zoetis Animal Health, Parsippany, NJ); metoclopramide (2 mg/kg/day CRI; Hospira, Lake Forest, IL); and ampicillin-sulbactam (30 mg/kg IV q8h; Pfizer, New York, NY).

The dog remained eupneic through the next morning, with a normalized iCa 0.93 mmol/L (RI: 0.91–1.32). Based on the clinical and laboratory improvements, the dog was discharged from the hospital later that day. The owners were instructed to administer calcitriol (14 ng/kg PO q12h; Hikma, Cherry Hill, NJ); calcium carbonate (120 mg/kg PO q12h; GSK, Durham, NC); carprofen (2.3 mg/kg PO q12h; Zoetis Animal Health, Parsippany, NJ); and trazodone (6.3 mg/kg PO q8h), in addition the previously prescribed amoxicillin-clavulanate and gabapentin. The dog was re-examined 4 days later. The dog was reported to have done well at home, without further episodes of respiratory difficulty, inspiratory stridor, or anxiety, and with a good appetite. A venous blood gas demonstrated a mild primary metabolic alkalosis [pH 7.49 (RI 7.3–7.48); *P*CO_2_ 43 mmHg (32–52); HCO_3_ 32.8 mmol/L (20.7–29.2)] and a normal ionized calcium 1.06 mmol/L (RI: 0.91–1.32). The dog has continued to do well in the subsequent 13 months following ethanol ablation of the parathyroid nodule without further episodes of respiratory distress.

## Discussion

This case report describes a dog with hyperparathyroidism that developed respiratory distress 72 h after ethanol ablation of a parathyroid nodule due to laryngospasm associated with hypocalcemia (4–7). The dog did not exhibit signs of respiratory abnormalities prior to the procedure or for the first 72 h after it, at which point hypocalcemia was noted concurrent to its respiratory signs. Correction of hypocalcemia, along with anxiolysis and oxygen supplementation, resulted in rapid and sustained improvement in clinical signs. Laryngospasm associated with hypocalcemia has been described in people, but to the authors' knowledge has not been described in dogs before (4–7). It is important to recognize that respiratory distress could be the only clinical sign of hypocalcemia in a patient following ethanol ablation of a parathyroid nodule.

Percutaneous ultrasound-guided ethanol ablation of parathyroid nodules achieves successful resolution of hypercalcemia in 95% of dogs with primary hyperparathyroidism (1, 3). The procedure is performed under anesthesia, where the ventral cervical region over the parathyroid nodule is clipped and aseptically prepped. Ultrasound is used to guide the insertion of a 27-gauge needle into the parathyroid nodule before injecting 96% ethanol (1, 2). The maximum volume of the mass is calculated as length × width × height, and the target volume of ethanol infusion is typically calculated as half the maximum volume of the mass. Evidence of diffusion of ethanol throughout the mass is observed, and then the needle is removed, and the patient recovers from anesthesia.

In people, adverse effects of ethanol ablation include incomplete necrosis of the tumor, hypocalcemia, and peri-glandular fibrosis (9). Reported complications of ethanol ablation in dogs include transient bark change, coughing, and dysphonia which can be attributed to ethanol leakage outside of the parathyroid nodule (2). Temporary recurrent laryngeal nerve injury has been reported to occur in 30% of people following ethanol ablation of parathyroid nodules (10), typically causing unilateral palsy of the nerv (11). The canine larynx consists of four cartilages surrounding the rima glottidis, consisting of the paired arytenoid cartilages, and unpaired thyroid cartilage, cricoid cartilage, and epiglottis (12). The vocal processes connect the vocal folds to the arytenoid cartilages, with the laryngeal saccules being rostrolateral to the vocal folds. Abduction and external rotation of the arytenoid cartilages, which allows opening of the larynx, requires contraction of the cricoarytenoideus dorsalis muscle. This muscle is innervated by the caudal laryngeal nerve, a branch of the recurrent laryngeal nerve. Given the proximity of the recurrent laryngeal nerve to the thyroid and parathyroid glands, laryngeal dysfunction secondary to chemical or traumatic injury after the ethanol ablation procedure is plausible. Iatrogenic damage to the recurrent laryngeal nerve leading to laryngeal dysfunction has also been described in dogs following ventral slot surgery (13) and endotracheal intubation (14).

In this specific case, the dog's clinical signs of respiratory distress, attributed to laryngospasm, responded rapidly to correction of ionized hypocalcemia with administration of intravenous calcium gluconate. Hypocalcemia can occur in up to 40% of dogs with hyperparathyroidism after parathyroidectomy (3) and 22% after ethanol ablation of parathyroid nodules (1), although only a small percentage of patients (10 and 3.7%, respectively) will become clinical for their hypocalcemia. Hypocalcemia most commonly manifests as neuromuscular signs in dogs, including tremors, muscle fasciculations, tetany, and less commonly seizures. Additional clinical signs include agitation, hyperthermia, hyperventilation, facial pruritus, and abdominal pain (16). The authors hypothesize that symptomatic hypocalcemia precipitated the upper respiratory crisis described in this case and might also explain the agitation and restlessness described by the owners. Other clinical signs of hypocalcemia were not observed in this dog, which could mean laryngospasm could be an isolated clinical sign, as has been described in people (4–7). Following this event, the dog was started on calcitriol and calcium carbonate, without subsequent recurrence of clinical signs. These therapies are usually initiated upon recognition of hypocalcemia with compatible clinical signs.

It is recommended that iCa be checked frequently post-operatively as the median time to ionized calcium nadir is ~48-h (3). For dogs with a pre-operative ionized calcium of >1.7 mmol/L, like the dog described in this case report, careful calcium monitoring is recommended since the risk of post-operative hypocalcemia is enhanced (3). In this specific case, iCa concentrations were measured on three occasions following ethanol ablation and showed a progressive decline over the initial 72 h period. An anal sac abscess was incidentally noted during this period, ~24 h before the onset of overt signs of respiratory distress. With hindsight, some of the dog's signs that were attributed to the pain and discomfort of the anal sac abscess might have been explained by hypocalcemia. Earlier administration of therapies to correct hypocalcemia might have circumvented the progression to respiratory distress in this case.

Alternative explanations for neuromuscular dysfunction in this specific case include hypomagnesemia, hypokalemia, and concurrent endocrinopathies. Regrettably, the magnesium concentration in this dog at the time of laryngospasm is not known with certainty. Prior to the ethanol ablation procedure, the dog had a normal magnesium concentration, therefore we consider it unlikely that hypomagnesemia was a likely contributor to its laryngeal dysfunction. In addition, the dog was normokalemic at the time of respiratory distress and had well-controlled hypercortisolism and a normal thyroxine concentration. Therefore, hypocalcemia remains the most likely explanation for the documented laryngospasm.

There are several limitations to the case description here that warrant discussion. While laryngeal dysfunction from temporary dysfunction of the recurrent laryngeal nerve and laryngospasm from hypocalcemia are plausible complications of ethanol ablation, the true prevalence of these issues is not known since laryngoscopy is not routinely performed after ethanol ablation. In this case, the dog was reported to show sustained clinical resolution following discharge from the hospital, without abnormal respiratory noise or subsequent upper airway crises, suggesting sustained correction of the issue at hand. A sedated laryngeal exam was not repeated however to confirm normalized laryngeal function.

In this specific case, electromyography could have been a helpful addition to characterize laryngeal function in situations with atypical clinical presentations (15).

Secondly, while medical record notes for this dog document normal cranial nerves, an anxious disposition, and strongly ambulatory status, more comprehensive neurological descriptions are not available. This provides limitations to the study as other subtle or transient neurological deficits may not have been observed or accounted for. It should also be noted that the laryngeal examination in this dog was conducted using a combination of butorphanol and alfaxalone. False positive results of laryngoscopy, where there is no arytenoid movement in an otherwise normal dog, may occur after the administration of certain anesthetic agents. Several studies have explored the impact of anesthetic drugs on laryngeal function during sedated laryngeal examinations (17–21). The dog in this case report was administered butorphanol, followed by alfaxalone to facilitate the sedated oral exam. Previous studies have indicated that alfaxalone can falsely reduce arytenoid opening and movement after its administration (19, 20), while other studies have found alfaxalone to be an acceptable alternative to propofol for laryngoscopy (17, 18). In this specific case, the dose of alfaxalone used (total dose of 0.5 mg/kg IV) was lower compared to the doses described to negatively affect arytenoid motion (19, 20) and enabled preservation of pharyngal and laryngeal function. The addition of doxapram, a centrally acting respiratory stimulant, could have been utilized however to further optimize laryngeal function in this case (19, 21). Ultrasonographic evaluation of arytenoid motion is possible in awake dogs and could offer an alternative to traditional laryngoscopy. This is an attractive option to remove the influence of anesthesia drugs on laryngeal function (22).

In conclusion, this case report describes the development of laryngospasm in a dog with hypocalcemia following ethanol ablation of a parathyroid gland nodule. This was determined based on compatible clinical signs and direct laryngoscopy performed under a light plane of anesthesia. Clinical signs resolved with correction of hypocalcemia, along with supplemental oxygen and anxiolysis. Sustained improvement was noted without recurrence of respiratory symptoms. Laryngospasm, secondary to hypocalcemia, has been described in people, but to the authors' knowledge has not been reported in dogs before. Focused respiratory monitoring and ionized calcium measurements following ethanol ablation procedures for hyperparathyroidism should be recommended in dogs.

## Data availability statement

The raw data supporting the conclusions of this article will be made available by the authors, without undue reservation.

## Ethics statement

Written informed consent was obtained from the participant/patient(s) for the publication of this case report.

## Author contributions

KR and AL: manuscript preparation. RG, LR-J, and KH: case management and manuscript preparation. YU: manuscript preparation. All authors contributed to the article and approved the submitted version.

## Supplementary material

Supplementary Video 1 Video showing laryngoscopy in a dog with hyperparathyroidism presented for evaluation of inspiratory distress and stridor 72 h after ethanol ablation of a parathyroid nodule. Laryngoscopy was performed under a light plane of anesthesia following the administration of butorphanol and alfaxalone. Reduced motion of both arytenoid cartilages was noted during inspiration, with complete closure occurring during expiration. These findings were most consistent with laryngospasm. The dog was hypocalcemic (ionized calcium 0.7 mmol/l, reference interval: 0.9–1.3 mmol/L).
